# Synthesized 18-lead electrocardiogram as routine myocardial ischemia detection in an emergency department: a preliminary evaluation in Europe

**DOI:** 10.1186/cc14241

**Published:** 2015-03-16

**Authors:** N Eppe, R Levy, M Vandoorslaert, B Kerzmann, J Rodrigues de Castro, T Sottiaux, JF Adam

**Affiliations:** 1 Clinique Notre-Dame de Grâce, Gosselies, Belgium

## Introduction

Standard 12-lead electrocardiogram (ECG) is, with biomarkers, the most accurate method in the diagnosis of acute coronary syndrome (ACS). However, posterior (V7-V8-V9) and right (V3R-V4R-V5R) derivations are not systematically performed due to the time-consuming procedure involved, despite major therapeutic implications (fluid loading instead of nitrates use in right ventricular involvement) and published guidelines [1]. Recently, an 18-lead ECG system, standard 12-lead ECG and six additional synthesized leads (assessing posterior and right ventricular areas) in only one recording procedure has been developed. The reliability of this material (ECG 2550; Nihon Kohden Co. Ltd, Japan) was already validated in this indication in an Asian population [2,3].

## Methods

We conducted a prospective, observational study with patients admitted to our emergency department (ED), during a 6-month period. Requirement for ECG was guided by physician's discretion according to patient's history. All patients with chest pain, dyspnea, palpitations, disturbance of consciousness, malaise or abdominal complaint underwent synthesized 18-lead ECG within 10 minutes of ED arrival. The aim of the study was to evaluate the effectiveness of the synthesized 18-lead ECG as an ischemia triage tool in the ED, and particularly the ability to early detect a right ventricular involvement.

## Results

Of the 3,835 nontraumatic patients treated in the ED, 3,196 were adults. In this adult population, 500 ECGs were performed in patients whose symptoms suggest ACS. The median age was 62.3 years and the sex ratio was 1.16. Clinical presentation was chest pain (31%), dyspnea (14%), palpitations (5%), disturbance of consciousness (3%) or others (47%). Fifty-six (11.2%) were diagnosed as ACS, including 20 ST-elevation myocardial infarction (STEMI), 28 non-STEMI and eight unstable angina. Of the 20 STEMI patients, eight (40%) and five (25%) were diagnosed as STEMI complicated by right ventricular and posterior wall ischemia respectively, which means that these complications could have been missed by standard 12-lead ECG.

## Conclusion

Eighteen-lead ECG with synthesized right-sided and posterior precordial leads was an efficient method to diagnose ACS in a Caucasian population within 10 minutes of ED arrival. It is particularly performant to detect right ventricular ischemia early, which can modify acute therapeutic strategy.
